# Supplementary material for: Unique Structural Features Relate to Evolutionary Adaptation of Cytochrome P450 in the Abyssal Zone
Source: Int J Mol Sci. 2025 Jun 13;26(12):5689. doi: 10.3390/ijms26125689 (PMC12192976; doi:10.3390/ijms26125689)
Supplement: Supplementary file 1 [file ijms-26-05689-s001.zip › ijms-3674443-supplementary.pdf]

Supplementary Materials

Unique structural features relate to evolutionary adaptation of cytochrome P450 in the abyssal zone

Tatiana Y. Hargrove[1], David C. Lamb[2], Zdzislaw Wawrzak[3], George Minasov[3], Jared V Goldstone[4], Steven L. Kelly[2], John Stegeman[4], and Galina I. Lepesheva*[,1,5]

[1]*Department of Biochemistry, Vanderbilt University School of Medicine, Nashville, TN, 37232, USA*

[2]*Faculty of Medicine, Health and Life Science, Swansea University, Swansea, SA2 8PP, UK*

[3]*Northwestern University, Argonne, IL, 60439, USA*

[4]*Biology Department, Woods Hole Oceanographic Institution, Woods Hole, Massachusetts 02543, USA*

[5]*Center for Structural Biology, Vanderbilt University, Nashville, Tennessee 37232, USA*.

[‡]*Center for Structural Biology, Vanderbilt University, Nashville, Tennessee 37232, USA.*

```
                                    *        20         *        40         *        60
Human                     :MLLLGLLQAGGSVLGQAMEKVTGG---NLLSMLLIACAFTLSLVYLIRLAAGH-LVQLPAGVKS:60
C armatus                 :~MAASLY---QMIFGDSGGMMSSSGGSSLTSVFLAASVVTLALGYLSKVLLQPTAS-SSGDQKC:59
Lota lota                 :~MAAPLY---KMFFGDTVGKMS-D---NLTSVFLAASVITLALGYLSKTMLQQSS--SN-SDRY:53
Arctogadus glacialis      :~MAAPLY---QMLFGDTVGKMS-D---NLTSVFLAVSVITLALGYLSKMMLQQST--SDPNVKY:54
Gadus chalcogrammus       :~MAAPLY---QMLFGDTVGKMS-D---NLTSVFLAVSVITLALGYLSKMMLQQST--SDPNVKY:54
Merluccius poli           :~MAAPSY---QTLFADAVGKVVSD---HLTSVLLAASLVTLALGYLSKVLLQQT----PPDLKY:53
Muraenolepis orangiensis  :MAAAPWY---QTMFGEAMGRLSSD---GLTSAVLAASVLALALGFLSKRLLQQAS--SSTDLKY:56
Danio rerio               :~~~MTILEVGSQLIESAV--L-QM---SLTSVLLTASVFTLTLGYFSKLLFTQHS---SEHTKY:52
Salmo trutta              :~MAMHLYQVSSMLLENTVGKM-SD---NLTSVVLAVSVITLTLGYISKLVLKQSQSSSEEHKKY:59
Acanthopagrus latus       :~MSLHLYEMSSKLFGETVGKV-NE---NLTSVVLAASLVTLIIGYVSKVLLKQSS--DSEDKKY:57
Larimichthys crocea       :~~~MHFYEMSSRLIGDTVSKV-TD---NVTTVVLAASVITLILGYVSKVLLRQSS--D-KDLKY:54
Channa argus              :~~~MQLYQLSSKLLGDTVGKM-ND---NLTSIVLAASALTLTLGYISKLLLRQSS--D-KDLKC:54
Lateolabrax maculatus     :~MSVLFYELSSRLLVDAVGKM-ND---NLTSVVLAASVITLTLGYVSNVLLRQPP--D-ADLKY:56
Xiphias gladius           :~MSMHFYQISSKLLGDTVGKM-ND---NLTSIVLAASVITLTLGYISKMLLRQSS--D-KDLKY:56
Pseudochaenichthys georg. :~MSVQFYEMGSRLFGDTVGKV-SD---NLTSVVLAASVITLALGYITKMLLKESP--D-TDLKY:56
Sebastes umbrosus         :~MAIHLYEMSSKLLGDTVGKM-SD---NLTSVVLAASVITLTLGYLSKMLLKQSS--D-SDLKY:56
Anarrhichthys ocellatus   :~MSFHLYEMSSKLLGDTVGTM-ND---NLTSVVLAASVVTLTLGYLSKLLLKPSS--D-NDLKY:56

                                *        80         *       100         *       120
Human                     :PPYIFSPIPFLGHAIAFGKSPIEFLENAYEKYGPVFSFTMVGKTFTYLLGSDAAALLFNSKNED:124
C. armatus                :PPYIPSRIPFLGHAVAFGKNPIEFLEKAYEKYGPVVSFTMVGKTFTFLLGSDAAALMFNSKNED:123
Lota lota                 :PPYIPSRIPFLGQAVAFGKSPIEFLENAYEKYGPVVSFTMVGKTFTFLMGSDAAALMFNSKNED:117
Arctogadus glacialis      :PPYIPSRIPFLGQAVAFGKNPIEFLEKAYEKYGSVVSFTMVGKTFTFLLGSDAASLMFNSKNED:118
Gadus chalcogrammus       :PPYIPSRIPFLGQAVAFGKSPIEFLEKAYEKYGSVVSFTMVGKTFTFLLGSDAASLMFNSKNED:118
Merluccius poli           :PPYIPSRIPFLGQAVAFGKSPIEFLEKAYEKYGPVVSFTMVGKTFTFLLGSDAAALMFNSKNED:117
Muraenolepis orangiensis  :PPYIPSRIPFLGQAVAFGKSPIEFLENAYEKYGPVVSFTMVGKTFTFLLGSDAAALMFNSKNED:120
Danio rerio               :PPHIPSSLPFLGQAVAFGRSPIEFLEKAYEQYGPVVSFTMVGKTFTYLLGSDAAALMFNSKNED:116
Salmo trutta              :PPYIPSSIPFLGHAIAFGKSPIEFLENAYEKYGPVVSFTMVGKTFTYLLGSEAATLMFNSKNED:123
Acanthopagrus latus       :PPFIPSSIPFLGHAIAFGKSPIEFLENAYEKYGPVFSFTMVGSTFTYLLGSEAATLMFNSKNDD:121
Larimichthys crocea       :PPYIPSSIPFLGHAIAFGKSPIEFLENAYEKYGPVFSFTMVGSTFTYLLGSDAAQLMFNSKNDD:118
Channa argus              :PPYIPSSIPFLGHAIAFGKSPIEFLENAYEKYGPVFSFTMVGKTFTYLLGSEAASLMFNSKNED:118
Lateolabrax maculatus     :PPYIPSSVPFLGHAIAFGKSPIEFLENAYEKYGPVFSFTMVGSTFTYLLGSDAATLMFNSKNED:120
Xiphias gladius           :PPYIPSSVPFLGHAIAFGKSPIEFLENAYEKYGPVFSFTMVGKTFTYLLGSEAATLLFNSKNED:120
Pseudochaenichthys georg. :PPYIPSSIPFLGHAIAFGKSPIEFLENAYEKYGPVFSFTMVGSTFTYLLGSEAATLMFNSKNED:120
Sebastes umbrosus         :PPYIPSSIPFLGHAIAFGKSPIEFLENAYEKYGPVFSFTMVGSTFTYLLGSEAATLMFNSKNED:120
Anarrhichthys ocellatus   :PPYIPSSIPFLGHAIAFGRSPIEFLEHAYEKYGPVFSFTMVGSTFTYLLGSEAATLMFNSKNED:120

                            *      140         *       160         *       180         *
Human                     :LNAEDVYSRLTTPVFGKGVAYDVPNPVFLEQKKMLKSGLNIAHFKQHVSIIEKETKEYFESWGE:188
C. armatus                :LSAEDVYSKITTPVFGKGVAYDVPNPIFLEQKKMLKTGLNIAQFKKHVPIIEQETEEYIKRWGD:187
Lota lota                 :LSAEDVYSKITTPVFGKGVAYDVPNPIFLEQKKMLKTGLNIAQFKKHVSIIENETEEYMKRWGD:181
Arctogadus glacialis      :LSAEDVYSKITTPVFGKGVAYDVPNPIFLEQKKMLKTGLNIAQFKKHVSLIEDETVEYMKRWGD:182
Gadus chalcogrammus       :LSAEDVYSKITTPVFGKGVAYDVPNPIFLEQKKMLKTGLNIAQFKKHVSLIEDETVEYMKRWGD:182
Merluccius poli           :LSAEDVYSKITTPVFGKGVAYDVPNPIFLEQKKMLKTGLNIAQFKKHVAIIEKETQEYIKRWGE:181
Muraenolepis orangiensis  :LSAEDVYSKITTPVFGKGVAYDVPNPIFLEQKKMLKTGLNIAQFKKHVTIIENETEEYMKRWGD:184
Danio rerio               :LNAEDVYARLTTPVFGKGVAYDVPNPLFLEQKKMLKTGLNIAQFKQHVEIIEEETKDYFRRWGE:180
Salmo trutta              :LNAEDVYSRLTTPVFGKGVAYDVPNHIFLEQKKMFKTGLNIAHFKQHVEIIEEETKEYFSRWGD:187
Acanthopagrus latus       :LNAEDVYSRLTTPVFGKGVAYDVPNHIFLEQKKMLKTGLNIARFREHVKVIEAETIEYFQRWGD:185
Larimichthys crocea       :LNAEDVYSRLTTPVFGKGVAYDVPNPVFLEQKKMLKTGLNIAHFREHVKIIEAETKEYFQRWGD:182
Channa argus              :LNAEDVYSRLTTPVFGKGVAYDVPNPIFLEQKKMLKSGLNIAHFKEHVKIIEAETIDYFQRWGD:182
Lateolabrax maculatus     :LNAEDVYSRLTTPVFGKGVAYDVPNPIFLEQKKMLKTGLNIAHFKQHVKVIEAETIEYFERWGD:184
Xiphias gladius           :LNAEDVYSRLTTPVFGKGVAYDVPNPIFLEQKKMLKTGLNIAHFKEHVKIIEAETVEYFQRWGD:184
Pseudochaenichthys georg. :LNAEDVYSRLTTPVFGKGVAYDVPNHIFMEQKKMLKTGLNIAHFKEHVKIIEAETIEYFQRWGD:184
Sebastes umbrosus         :LNAEDVYSRLTTPVFGKGVAYDVPNPIFLEQKKMLKTGLSIAHFKEHMKIIEAETIEYFQRWGD:184
Anarrhichthys ocellatus   :LNAEDVYSRLTTPVFGKGVAYDVPNPIFLEQKKMLKTGLNIAHFKEHVKIIEAETIKYFQRWGD:184
```

```
                                      200         *         220         *         240         *
Human                         :SGEKNVFEALSELIILTASHCLHGKEIRSQLNE-KVAQLYADLDGGFSHAAWLLPGWLPLPSFR:251
C. armatus                    :SGETNLFVAMSELIILTASSCLHGKEIRSLLNE-KVAQLYCDLDGGFSHEAWLLPSWLPLPSFR:250
Lota lota                     :SGETNLFVALSELIILTASSCLHGKEIRSMLDE-KVAQLYCDLDGGFSHEAWLLPSWLPLPSFR:244
Arctogadus glacialis          :SGETNLFVALSELIILTASSCLHGKEIRSMLDE-KVAQLYCDLDGGFSHEAWLLPSWLPLPSFR:245
Gadus chalcogrammus           :SGETNLFVALSELIILTASSCLHGKEIRSMLDE-KVAQLYCDLDGGFSHEAWLLPSWLPLPSFR:245
Merluccius poli               :SGETNLFVALSELIILTASSCLHGKEIRSMLDE-KVAQLYYDLDGGFSHEAWLLPSWVPLPSFR:244
Muraenolepis orangiensis      :SGETNLFVALSELIILTASSCLHGKEIRSMLNE-KVAQLYCDLDGGFSHEAWLLPSWLPLPSFR:247
Danio_rerio                   :SGERNLFDALSELIILTASRCLHGCEIRSLLDE-RVAQLYADLDGGFTHAAWLLPGWLPLPSFR:243
Salmo_trutta                  :SGEQNLFEALSELIILTASACLHGKEIRSMLDE-KVAQLYADLDGGFSHAAWLLPGWLPLPSFR:250
Acanthopagrus latus           :SGEKNLFEALSELIILTASSCLHGKEIRSMLNE-RVAQLYADLDGGFSHAAWLLPGWLPLPSFR:248
Larimichthys crocea           :SGERNLFEALSELIILTASSCLHGKEIRSMLNE-RVAQLYADLDGGFSHAAWLLPGWLPLPSFR:245
Channa argus                  :SGERNLFEALSELIILTASSCLHGKEIRSMLNE-QVAQLYADLDGGFTHAAWLLPGWLPLPSFR:245
Lateolabrax maculatus         :SGEKNLFEALSELIILTASSCLHGKEIRSMLDE-RVAQLYADLDGGFSHAAWLLPGWLPLPSFR:247
Xiphias gladius               :SGERNLFEALSELIILTASSCLHGKEIRGMLNE-RVAQLYADLDGGFSHAAWLLPGWLPLPSFR:247
Pseudochaenichthys georg.     :SGEKNLFEALSELIILTASSCLHGKEIRSMMNE-HVAQLYADLDGGFSHAAWLLPGWLPLPSFR:247
Sebastes umbrosus             :SGEKNLFEALSELIIMTASSCLHGKEIRSMMNE-GVAQLYADLDGGFSHAAWLLPGWLPLPSFR:247
Anarrhichthys ocellatus       :SGERNLFEALSELIILTASSCLHGKEIRSMLNE-RVAQLYADLDGGFSHAAWLLPGWLPLPSFR:247

                                260         *         280         *         300         *         320
Human                         :RRDRAHREIKDIFYKAIQKRRQSQEKIDDILQTLLDATYKDGRPLTDDEVAGMLIGLLLAGQHT:315
C. armatus                    :RRDRAHREIKNMFYTAIQKRRTSNEKVDDFLQTLIDATYKDGHSLSDDEIAGLLIGLLLAGQHT:314
Lota lota                     :RRDRAHREIKSMFFKAIQKRRNSSEKVDDFLQTLIDATYKDGHTLSDDEIAGLLIGLLLAGQHT:308
Arctogadus glacialis          :KRDRAHREIKSMFFKAIQKRRNSSEKGDDFLQTLIDATYKDGHTLSDDEISGLLIGLLLAGQHT:309
Gadus chalcogrammus           :KRDRAHREIKSMFFKAIQKRRNSSEKGDDFLQTLIDATYKDGHTLSDDEISGLLIGLLLAGQHT:309
Merluccius poli               :RRDRAHREIKNMFFKAIEKRRTSKERVDDFLQTLIDATYKDGHSLSDDEIAGLLIGLLLAGQHT:308
Muraenolepis orangiensis      :RRDRAHREIKNIFFKAIQKRRTSSEKVDDFLQTLIDATYKDGHALSDDEIAGLLIGLLLAGQHT:311
Danio_rerio                   :RRDRAHLEIKKIFYNVIKKRREDTEKHDDILQTLIDATYKDGRPLSDDEIAGMLIGLLLAGQHT:307
Salmo_trutta                  :RRDRAHREIKNIFYKVTQKRRSSGEKVDDMLQTLIDATYKDGRPLNDDEIAGMLIGLLLAGQHT:314
Acanthopagrus latus           :KRDRAHREIKNIFFKVIQKRRQSTEKVDDILQTLVDATYKNGRPLNDDEIAGMLIGLLLAGQHT:312
Larimichthys crocea           :KRDRAHREIKNIFFKVTQKRRQSGEKVDDILQTLIDATYKDGRPLNDNEIAGMLIGLLLAGQHT:309
Channa argus                  :KRDRAHREIKNIFFKVIQKRRRSGEKVDDILQTLIDATYKDGRPLSDNEIAGMLIGLLLAGQHT:309
Lateolabrax maculatus         :KRDRAHREIKNIFFKVIQKRRRSGEKVDDILQTLIDATYKDGRPLSDNEIAGMLIGLLLAGQHT:311
Xiphias gladius               :KRDNAHREIKNIFFKVIQKRRRSGEKVDDILQTLIDATYKDGQPLDDNEIAGMLIGLLLAGQHT:311
Pseudochaenichthys georg.     :KRDNAHREIKNIFSKVTQKRRQSGEKVDDILQTLIDATYKDGRPLNDSEIAGMLIGLLLAGQHT:311
Sebastes umbrosus             :KRDRAHREIKNIFFKVIQKRRQSGEKVDDMLQTLVDATYKDGQPLNNDEIAGMLIGLLLAGQHT:311
Anarrhichthys ocellatus       :KRDRAHTEIKNIFFKVIQKRRQSEEKVDDMLQTLVDATYKDGRPLNDNEISGMLIGLLLAGQHT:311

                                         *         340         *         360         *         380
Human                         :SSTTSAWMGFFLARDKTLQKKCYLEQKTVCGENLPPLTYDQLKDLNLLDRCIKETLRLRPPIMI:379
C. armatus                    :SSTTSSWMSFFLARDKQLQERCLAEQKAVCGEDLPPLDFDQLKELSLLDRCLKETLRLRPPIMT:378
Lota lota                     :SSTTSAWMTFFLARDKQLQERCYAEQKAVCGDDLPPLEFDQLKDLGLLDRCVKETLRLRPPIMT:372
Arctogadus glacialis          :SSTTSAWMTFFLARDKELQERCYAEQKAVCGEDLPPLEFDQLKDLGLLDRCVKETLRLRPPIMT:373
Gadus chalcogrammus           :SSTTSAWMTFFLARDKELQERCYAEQKAVCGEDLPPLEFDQLKDLGLLDRCVKETLRLRPPIMT:373
Merluccius poli               :SSTTSSWMTFFLARDKKLQERCYAEQKAVCGDDLPPLEFDQLKDLSLLDRCVKETLRLRPPIMT:372
Muraenolepis orangiensis      :SSTTSTWMTFFLARDKQLQERCHAEQKAVCGDELPPLEFDQLKDLGLLDRCVKETLRLRPPIMT:375
Danio rerio                   :SSTTSAWMGFFLARDRALQERCYSEQKSVCGEELPPLHYDQLKDLSLLDRCLKETLRLRPPIMT:371
Salmo trutta                  :SSTTSSWLGFFLGKDKALQDRCYAEQKTACGEDLPPLNFDQLKDLTLLDRCLKETLRLRPPIMT:378
Acanthopagrus latus           :SSTTSAWMGFFMAKDRSLQDRCYAEQKAVCGEALPPLDFDQLKDLSLLDRCLKETLRLRPPIMT:376
Larimichthys crocea           :SSTTSSWMGFFMARDKSLQERCYAEQKAACGEDLPPLDYDQLKDLNLLERCLKETLRLRPPIMT:373
Channa argus                  :SSTTSAWMGFFLARDKDLQQRCYAEQKTVCGEDLPPLDFDQLKDLSLLERCLKETLRLRPPIMT:373
Lateolabrax maculatus         :SSTTSAWMGFFLARDKALQERCCAEQRAVCGEDLPPLDFDQLKDLSLLERCLKETLRLRPPIMT:375
Xiphias gladius               :SSTTSAWMGFFLARDKALQERCYAEQRAVCGEDLPPLDFDQLKDLSLLERCLKETLRLRPPIMT:375
Pseudochaenichthys georg.     :SSTTSAWMGFFMAKDKALQEKCYAEQKAVCGEDLPALDLDQLKDLSLLERCLKETLRLRPPIMT:375
Sebastes umbrosus             :SSTTSAWLGFFLARDKALQERCYAEQRAVCGEELPPLDFDQLKDLSLLERCLKETLRLRPPIMT:375
Anarrhichthys ocellatus       :SSTTSAWMGFFLARDKALQERCYAEQRAVCGEGL-ELDFDQLKDLSLLERCLKETLRLRPPIMT:374
```

```
                                   *       400          *         420          *       440
Human                        :MMRMARTPQTVAGYTIPPGHQVCVSPTVNQRLKDSWVERLDFNPDRYLQDNPASGEKFAYVPFG:443
C. armatus                   :MMRMARSPQCVAGYTIPVGHQVCVSPTVNQRLPDSWTERDEFNPDRYLTDNAATGEKFAYVPFG:442
Lota lota                    :MMRMARSPQCVAGYTIPVGHQVCVSPTVNQRLPDTWTERDQFTPDRFLNENAASGEKFSYVPFG:436
Arctogadus glacialis         :MMRMARSPQSVAGYTIPAGHQVCVSPTVNQRLPDTWTERDQFSPDRFLSESAASGEKFAYVPFG:437
Gadus chalcogrammus          :MMRMARSPQSVAGYTIPAGHQVCVSPTVNQRLPDTWTERDQFSPDRFLSESAASGEKFAYVPFG:437
Merluccius poli              :MMRMARSPQRVAGYTIPVGHQVCVSPTVNQRLPDTWTERDDFNPDRFLSDNAASGEKFAYVPFG:436
Muraenolepis orangiensis     :MMRLARSPQSVAGYTIPAGHQVCVSPTVNQRLPDTWTERHEFNPERFLTDNAASGDKFSYVPFG:439
Danio rerio                  :MMRMAKTPQKVGEYTIPPGHQVCVSPTVNHRLQDTWAERLDFDPDRYLHDNPAAGEKFAYIPFG:435
Salmo trutta                 :MMRMARSPQTVAGYTIPAGHQVCVSPTVNHRLKDTWTERMEFRPDRYLNDNPAAVEKFAYVPFG:442
Acanthopagrus latus          :MMRMARSPQTAAGYTIPVGHQVCVSPTVNHRLYDTWNERMEFNPDRYLNDNPAAGEKFAYVPFG:440
Larimichthys crocea          :MMRMARSPQTAAGYTIPVGHQVCVSPTVNHRLCDTWDKRLEFNPDRYLDENLAAGEKFAYVPFG:437
Channa argus                 :MMRMARSPQTAAGYTIPVGHQVCVSPTVNHRLHDTWVERMEFNPERYIGDNPAAGEKFAYVPFG:437
Lateolabrax maculatus        :MMRMARSPQTAAGYTIPVGHQVCVSPTVNHRLHDTWAERMEFNPDRYLNDNPAAGEKFAYVPFG:439
Xiphias gladius              :MMRMARSPQTAAGYTIPVGHQVCVSPTVNHRLHDTWAERMEFNPDRYLNDNPAAGEKFAYVPFG:439
Pseudochaenichthys georg.    :MMRMARSLQTAAGYTIPVGHQVCVSPTVNQRLQDTWDERMEFSPDRYLNDNPAAGEKFAYVPFG:439
Sebastes umbrosus            :MMRMARSPQSAAGYTIPVGHQVCVSPTVNQRLQDTWVERMEFSPDRYLNDNPAAGEKFAYVPFG:439
Anarrhichthys ocellatus      :MMRMARSPQTAAGYTIPVGHQVCVSPTVNQRLQDTWVERMEFSPDRYLNDNPAAGEKFAYVPFG:438
```

```
                               *        460          *         480          *        500          *
Human                        :AGRHRCIGENFAYVQIKTIWSTMLRLYEFDLIDGYFPTVNYTTMIHTPENPVIRYKRRSK~~~~:503
C. armatus                   :AGRHRCIGEGFAYVQLKTILSTLIRKFEFDLVDGYFPTINFTTMIHTPNNPVIRYRSRTH~~~~:502
Lota lota                    :AGRHRCIGENFAYVQLKTILSTMMRKYEFDLVNGYFPTINFTTMIHTPHNPVIRYRSRTH~~~~:496
Arctogadus glacialis         :AGRHRCIGENFAYVQLKTILSTMMRTYEFDLVDGYFPTINFTTMIHTPHNPVIRYRSRAH~~~~:497
Gadus chalcogrammus          :AGRHRCIGENFAYVQLKTILSTMMRAYEFDLVDGYFPTINFTTMIHTPHNPVIRYRSRAH~~~~:497
Merluccius poli              :AGRHRCIGENFAYVQLKTILSTMTRKYEFDLVDGYFPSINFTTMIHTPHNPVIRYKSRTP~~~~:496
Muraenolepis orangiensis     :AGRHRCIGENFAYVQLKTILSTMVRKYQFDLVDGYFPTINFTTMIHTPHNPVIRYKSRTH~~~~:499
Danio rerio                  :AGRHRCIGENFAYVQIKTIWSTLIRMFDFELVDGHFPPVNYTTMIHTPHNPIIRYTRRNTQPQQ:499
Salmo trutta                 :AGRHRCIGENFAYVQIKTIWSTMLRLYEFNLVDGYFPTINYTTMIHTPHNPVIRYKRRQH~~~~:502
Acanthopagrus latus          :AGRHRCIGENFAYVQIKTIWSTLLRMYDFDLVDGYFPTINYTTMIHTPHNPVIRYKKRIQ~~~~:500
Larimichthys crocea          :AGRHRCIGENFAYVQIKTIWSTLLRMYDFDLVDGYFPTINYTTMIHTPNNPVIRYKKRTQ~~~~:497
Channa argus                 :AGRHRCIGENFAYVQIKTIWSTLLRMYEFNLVDGYFPTINYTTMIHTPHNPFIRYKKRKQ~~~~:497
Lateolabrax maculatus        :AGRHRCIGENFAYVQIKTIWSTLLRMYDFDLVDGYFPTINYTTMIHTPHNPVIRYKRRKP~~~~:499
Xiphias gladius              :AGRHRCIGENFAYVQIKTIWSTLLRMYDFDLVDGYFPTINYTTMIHTPHTPVIRYKRRKQ~~~~:499
Pseudochaenichthys georg.    :AGRHRCIGENFAYVQIKTIWSTLLRMYDFDLVDGYFPTINYTTMIHTPHNPIIKYKRRAQ~~~~:499
Sebastes umbrosus            :AGRHRCIGENFAYVQIKTIWSTMLRMYEFDLVDGYFPTINYTTMIHTPHNPIIRYKRRKQ~~~~:499
Anarrhichthys ocellatus      :AGRHRCIGENFAYVQIKTIWSTLLRMYHFDLVDGYFPTINYTTMIHTPHNPVIRYQRRKQ~~~~:498
```

Figure S1. Multiple sequence alignment of CYP51 proteins from fish and human.

Coryphaenoides armatus: XQS36516.1 (800 - 5,500 m); *Lota lota*: CAL8298409.1 (below 300 m); *Arctogadus glacialis:* CAL8365208.1 (up to 1000 m, frequently under ice); *Gadus chalcogrammus:* XP_056438121 (up to 1,300 m); *Merluccius polli*: KAK0136477 (50-910 m); *Muraenolepis orangiensis:* KAJ3609750 (135 - 860 m); *Danio rerio:* NP_001001730; *Salmo trutta:* XP_029591193; *Acanthopagrus latus:* XP_036948155; *Larimichthys crocea:* XP_010739985; *Channa argus:* KAF3707363; *Lateolabrax maculatus:* QQL94751; *Xiphias gladius:* XP_039976906; *Pseudochaenichthys georgianus:* XP_033950401; *Sebastes umbrosus:* XP_037650187; *Anarrhichthys ocellatus:* XP_031722207. The residues that in the *C. armatus* CYP51 FG arm and β4 hairpin are different from the corresponding residues in human CYP51 are colored blue and purple, respectively.

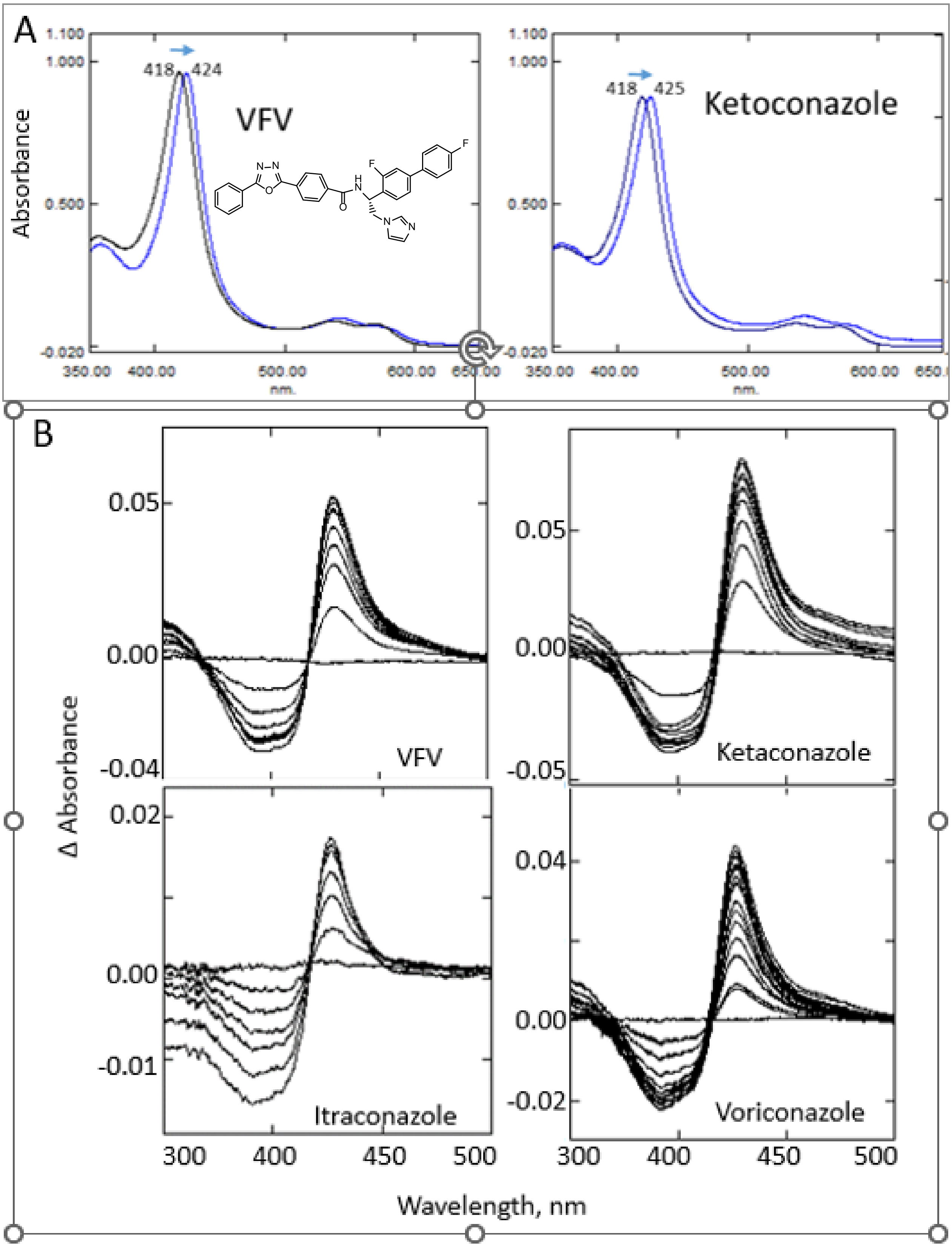

Figure S2. Spectral changes in *C. armatus* CYP51 in response to the addition of azoles. A. Absolute absorbance spectra. Black line - ligand-free, blue line - azole-bound protein sample. P450 concentration ~8 µM. **B**. Difference absorbance spectra upon titration (type II spectral response). P450 concentration 0.5 µM, titration step 0.1 µM.

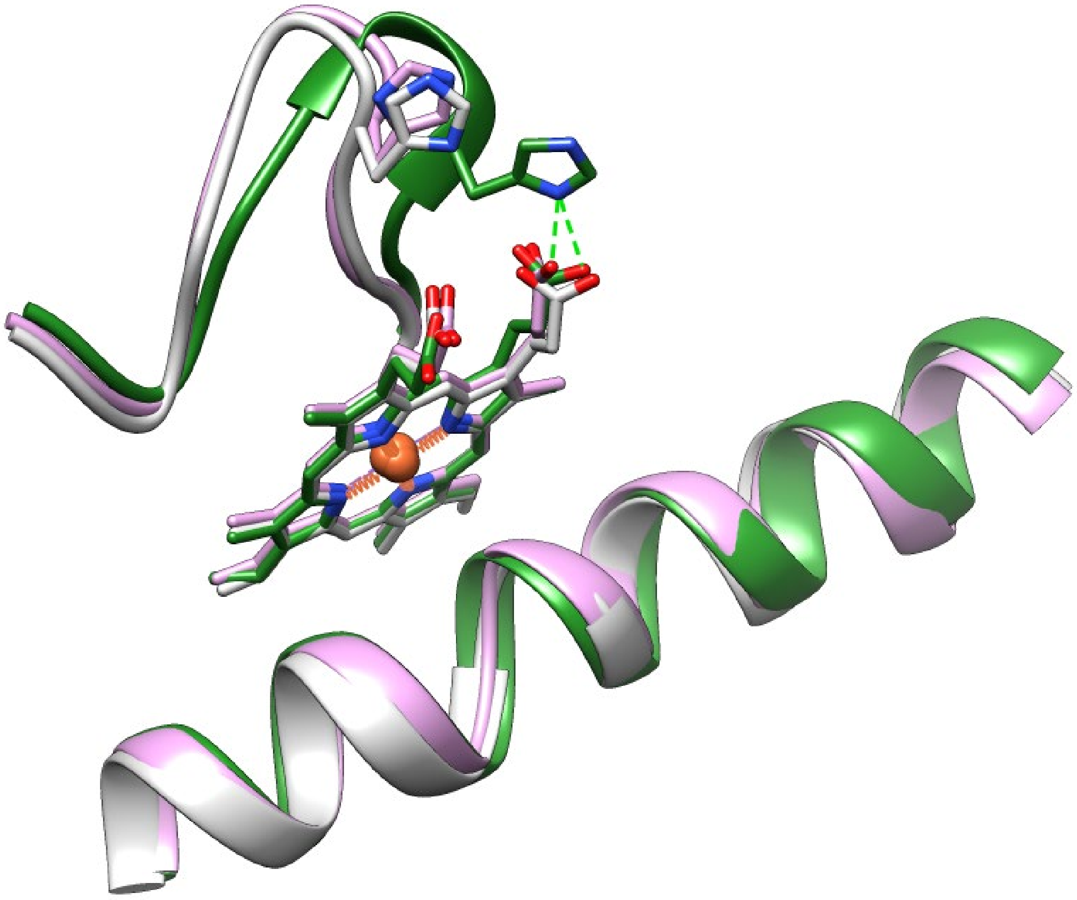

Figure S3. Helix I and the heme bulge in the AlphaFold model of ligand-free human CYP51, AF-Q16850-F1 (green). Superimposed with the structures of human CYP51 (8SBI, plum) and *C. armatus* CYP51 (9BAT, grey).

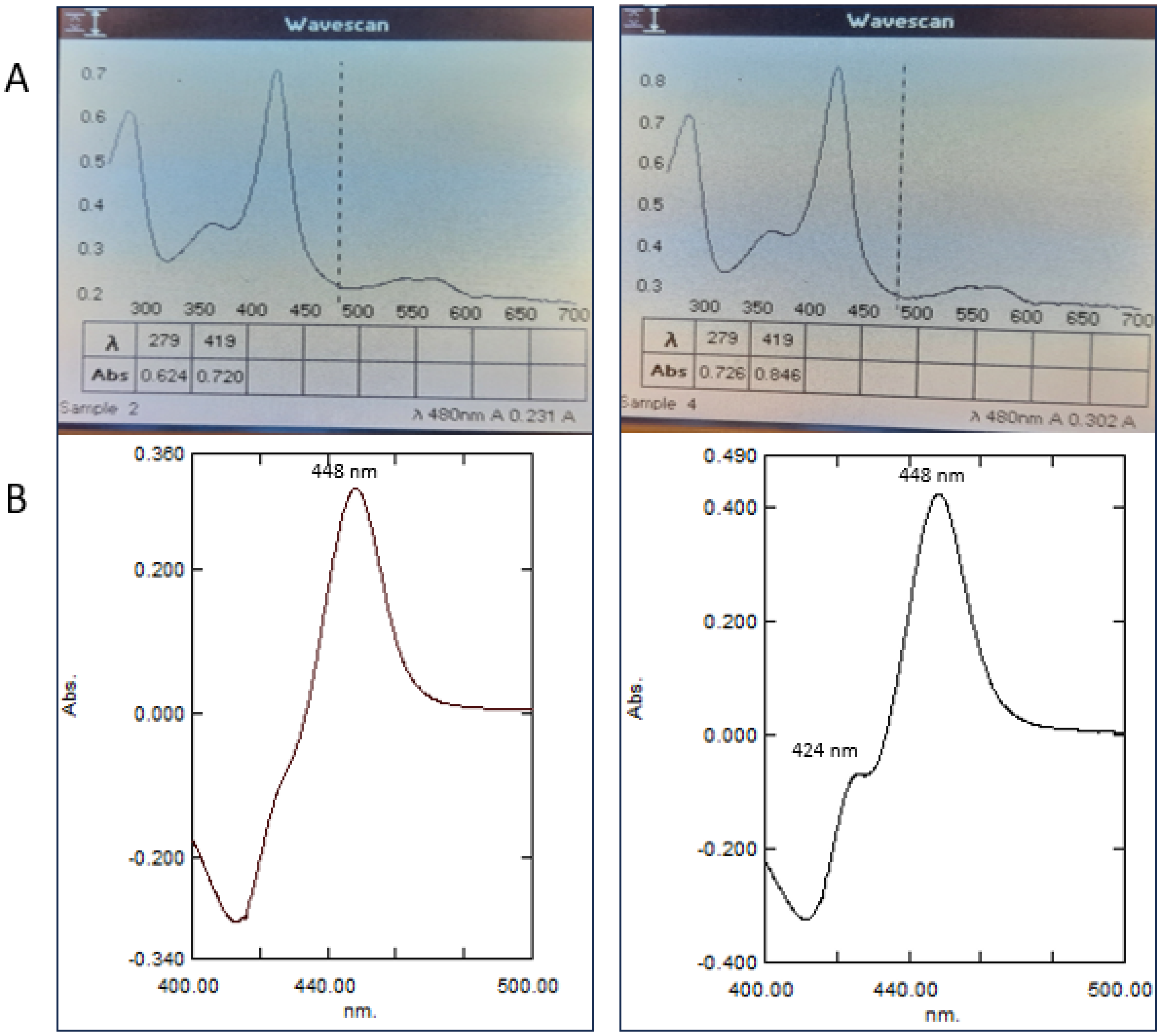

Figure S4. Absolute (**A)** and CO-binding difference **(B**) absorption spectra of the dissolved *C. armatus* CYP51 crystals (left panel), compared to the corresponding spectra of the protein sample used for crystallization (right panel).

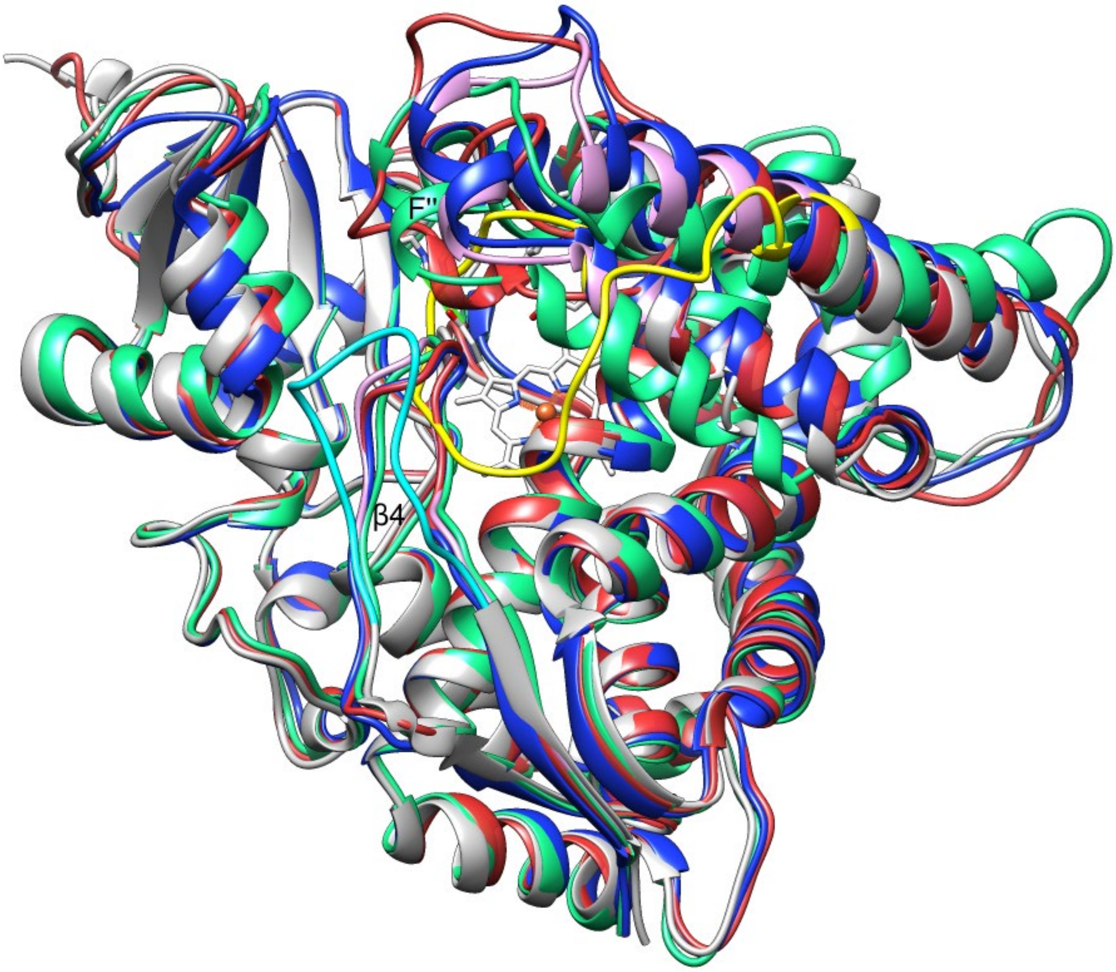

Figure S5. Superimposition of 9BAT and 8SBI with other three human CYP51 structures from PDB further supports the notion about high structural flexibility of CYP51s from vertebrates. The ribbon of 3JUV (ligand-free) is red, the ribbon of 4UHI (VFV-bound) is blue, and the ribbon of 6UEZ (lanosterol-bound) is green**.** The ligands are deleted for clarity. Orientation is the same as in Figure 5.
